# Hypoxia-Inducible Factor-1α Target Genes Contribute to Retinal Neuroprotection

**DOI:** 10.3389/fncel.2017.00020

**Published:** 2017-02-27

**Authors:** Lin Cheng, Honghua Yu, Naihong Yan, Kunbei Lai, Mengqing Xiang

**Affiliations:** ^1^State Key Laboratory of Ophthalmology, Zhongshan Ophthalmic Center, Sun Yat-sen UniversityGuangzhou, China; ^2^Department of Ophthalmology, General Hospital of Guangzhou Military Command of PLAGuangzhou, China; ^3^Department of Ophthalmology and Ophthalmic Laboratories, State Key Laboratory of Biotherapy and Cancer Center, West China Hospital, Sichuan UniversityChengdu, China; ^4^Center for Advanced Biotechnology and Medicine and Department of Pediatrics, Rutgers University-Robert Wood Johnson Medical SchoolPiscataway, NJ, USA

**Keywords:** HIF-1α, hypoxia preconditioning, retina, neuroprotection, retinal degeneration

## Abstract

Hypoxia-inducible factor (HIF) is a transcription factor that facilitates cellular adaptation to hypoxia and ischemia. Long-standing evidence suggests that one isotype of HIF, HIF-1α, is involved in the pathogenesis of various solid tumors and cardiac diseases. However, the role of HIF-1α in retina remains poorly understood. HIF-1α has been recognized as neuroprotective in cerebral ischemia in the past two decades. Additionally, an increasing number of studies has shown that HIF-1α and its target genes contribute to retinal neuroprotection. This review will focus on recent advances in the studies of HIF-1α and its target genes that contribute to retinal neuroprotection. A thorough understanding of the function of HIF-1α and its target genes may lead to identification of novel therapeutic targets for treating degenerative retinal diseases including glaucoma, age-related macular degeneration, diabetic retinopathy, and retinal vein occlusions.

## Introduction

**Figure 1:**
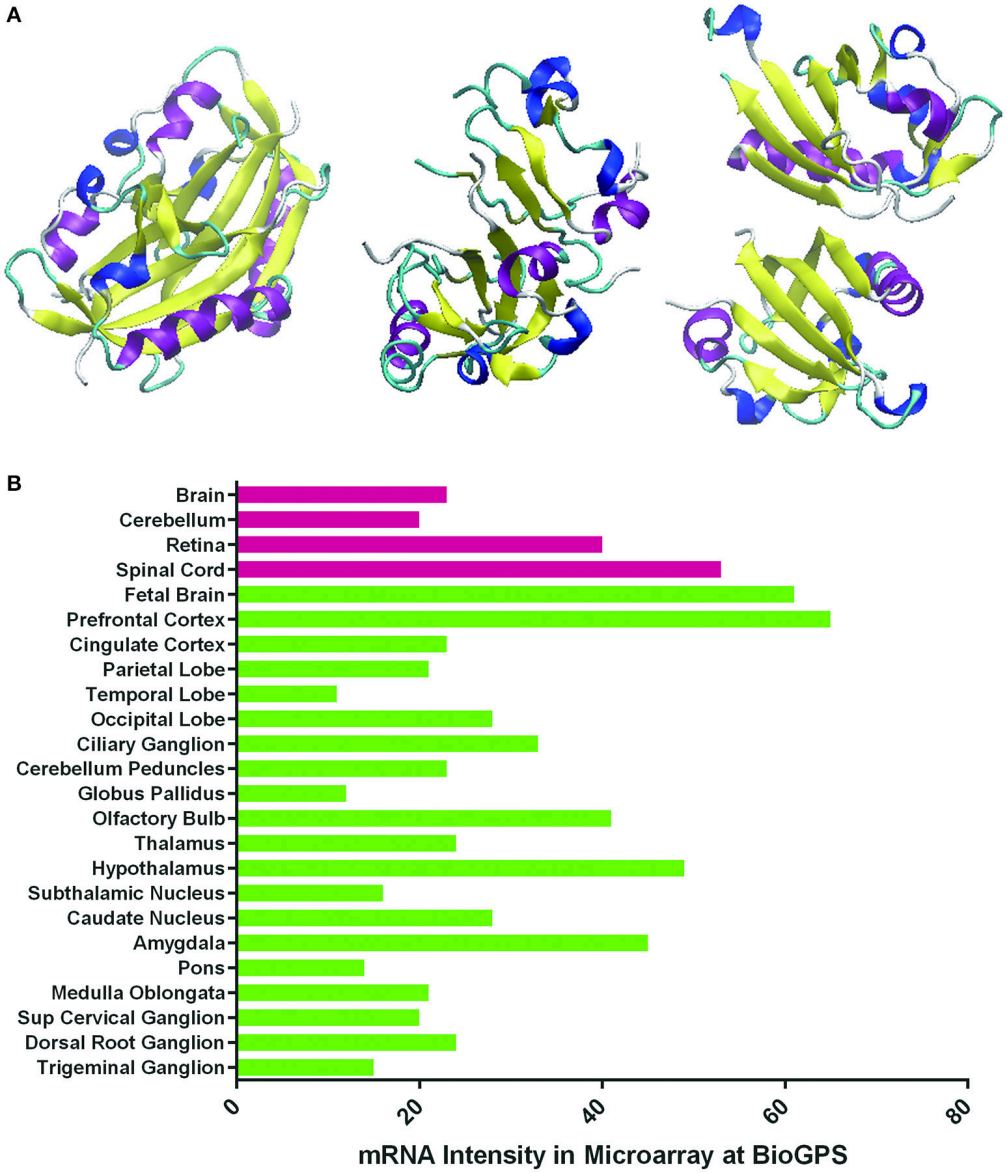
**Three-dimensional structures of HIF's alpha subunits and HIF-1α expression levels in normal human nervous tissues. (A)** Schematic representation of the crystal structures of HIF-1α, HIF-2α, and HIF-3α proteins reported at the Protein Data Bank (PDB) with PDB ID 4H6J, 4GHI, and 4WN5, respectively (http://www.rcsb.org/pdb/home/home.do). Different structural parts are highlighted in the following colors: magenta: α-helix; yellow: residue in isolated β-bridge; blue: loop; cyan: hydrogen bounded turn; white: bend. They contain the N-terminus, central region and C-terminus. **(B)** HIF-1α mRNA expression levels in normal human nervous tissues (normalized intensities in microarray) reported in Genecards (http://www.genecards.org).

**Figure 2:**
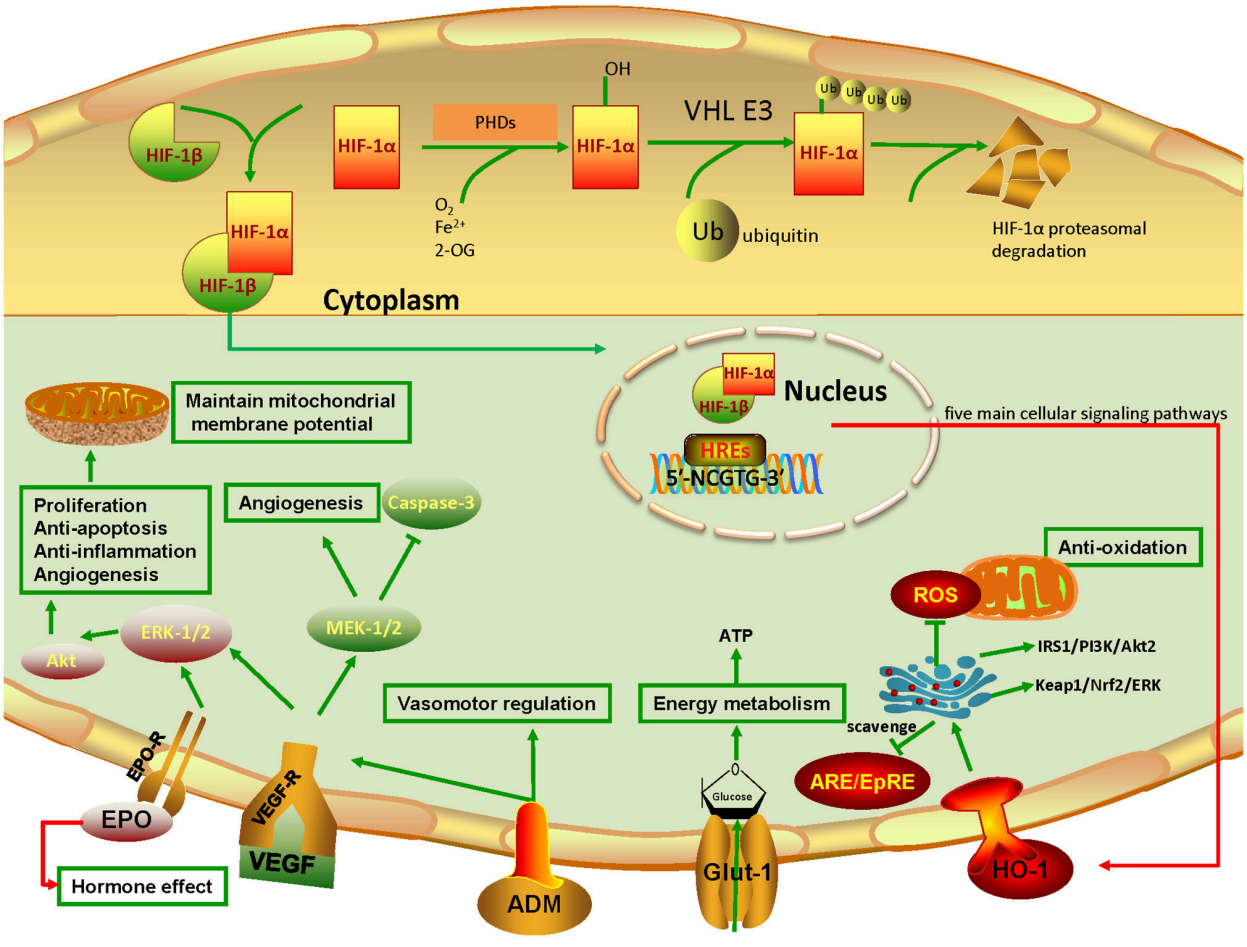
**Pathways of HIF-1α and its target genes involved in retinal neuroprotection**. The upper panel in yellow background is the schematic representation of HIF-1α degradation under normoxia. Note that the undegraded HIF-1α binds with HIF-1β to form the HIF-1α/β complex. The complex binds to HIF-responsive elements (HREs) in promoters that contain the sequence motif 5′-NCGTG-3′ and triggers transcription of more than 100 downstream genes. VHL: von Hippel–Lindau tumor suppressor protein (E3 ubiquitin protein ligase). The lower panel in pale blue color represents the HIF-1α target genes and their acting pathways involved in retinal neuroprotection under hypoxia. Note that five main cellular signaling pathways mediating the effect of neuroprotection are highlighted. Firstly, EPO binds to EPO-R to promote ERK-1/2 signaling, and then activate the Akt pathway, resulting in cell proliferation, anti-apoptosis, anti-inflammation, and angiogenesis. Also, it can maintain mitochondrial membrane potential to prevent mitochondrial alteration. In particular, EPO can be pumped outside the cytoplasm, which leads to the autocrine and paracrine effects that further exert retinal neuroprotection. Secondly, VEGF, which binds to VEGF-R, can achieve the same effects as EPO through activating ERK-1/2 signaling. Simultaneously, it enhances the MEK-1/2 pathway, promoting angiogenesis and inhibiting caspase-3 to constrain cell death. Thirdly, the secreted multifunctional peptide ADM mainly plays roles in vasomotor regulation, and acts together with VEGF to promote angiogenesis. Fourthly, Glut-1 transports glucose to the cytoplasm, allowing normal metabolic activity. Fifthly, HO-1 is degraded by ARE/EpRE elements. While under hypoxia, HO-1 blunts reactive oxygen species (ROS) production and the toxic effect on mitochondria. More importantly, HO-1 retards retinal injury through the IRS1/PI3K/Akt2 and Keap1/Nrf2 pathways, which further activate mTOR, upregulate anti-apoptotic proteins, and eliminate ROS.

Hypoxia or ischemia stress is generally harmful for the organism but may become beneficial under certain circumstances. Reduced oxygenation leads to induction of a number of hypoxia-responsive genes. The known hypoxia-inducible factor (HIF) targets such as erythropoietin (EPO), vascular endothelial growth factor (VEGF), heme oxygenase-1 (HO-1), adrenomedullin (ADM), glucose transporter-1 (Glut-1), etc., exert neuroprotective effects on the central nervous system (CNS; Harten et al., 2010). For instance, hypoxia preconditioning has been shown to increase resistance of harmful insults by the brain (Kitagawa et al., 1990; Glazier et al., 1994; Gidday, 2006). Unlike in the brain, however, knowledge about the neuroprotective effects of HIF targets in the retina is limited. The roles of HIF targets in retinal neurogenesis and function are not well-defined and there is no consensus on their actual functions in the retina. Retinal degeneration diseases are the leading causes of blindness worldwide and some of them are closely related to ischemia (Song et al., 2003; Zheng et al., 2007; Fulton et al., 2009). Retinal hypoxia is known as the common pathogenic condition leading to vision loss, including in diseases like glaucoma, age-related macular degeneration (AMD), diabetic retinopathy (DR), retinal vein occlusion (RVO), and some retinal degeneration secondary to stroke or Alzheimer's disease (Blanks et al., 1989; Katz et al., 1989). One isotype of HIF, HIF-1α, is abundantly expressed in the retina (Figure 1B; Zhu et al., 2013). Under normoxia, HIF-1α protein is rapidly degraded and its expression is hardly detectable in the adult retinal tissue (Jewell et al., 2001). Under hypoxia stress, however, HIF-1α expression is upregulated and the protein is translocated to the nucleus to stimulate the expression of its downstream targets (Figure 2). These downstream targets such as EPO and VEGF, which are produced in a relatively low level to stabilize the normal development or integrity of retina, are increased substantially under hypoxia to accommodate these alterations. When overexpressed, these target proteins may cause some ocular diseases. On the other hand, the HIF-1α target genes such as EPO, HO-1, and Glut-1, have the properties to promote neurogenesis themselves. Therefore, unraveling the neuroprotective network of HIF-1α target genes in the retina is important for understanding the mechanisms underlying ocular diseases.

## HIF-1α and its target genes are involved in the pathophysiological roles of retina

In humans, HIF family members include HIF-1, HIF-2, and HIF-3. They have different roles in hypoxia. HIF-1 triggers the expression of several genes to promote survival in hypoxia. HIF-2 regulates other functions such as erythropoietin production in normoxia or hypoxia (Eckardt and Kurtz, 2005) and high-altitude adaptation (Yi et al., 2010; Hanaoka et al., 2012). HIF-3 is considered to be a negative regulator of hypoxia-inducible gene expression (Augstein et al., 2011; Heikkila et al., 2011; Ando et al., 2013). The structures of HIFs and HIF-1α expression in normal human nervous tissues are shown in Figure 1. HIF is composed of an alpha (α) and a beta (β) subunit. The α subunit belongs to the helix-loop-helix (bHLH) family of transcription factors. Under normoxia, it is hydroxylated at specific proline residues by HIF prolyl-hydroxylases (PHD), allowing its recognition and ubiquitination by the von Hippel-Lindau E3 ubiquitin ligase (VHL E3), which labels it for rapid degradation by the proteasome (Maxwell et al., 1999). The β subunit is a constitutively-expressed aryl hydrocarbon receptor nuclear translocator (ARNT). HIF α and β are constantly being produced and degraded at aerobic conditions. While under hypoxemia in the retina, only HIF-1α persists and the HIF-1α/β complex is translocated to the nucleus, where it binds to HIF-responsive elements (HREs) and induces the transcription of its target genes involved in oxygen delivery and energy metabolism (Figure 2). The target genes regulated by HIF-1α are mainly EPO, VEGF, HO-1, ADM, and Glut-1.

EPO is the first identified gene related to hypoxia which in turn led to the discovery of HIF (Semenza and Wang, 1992). It is a glycoprotein hormone predominately secreted by kidney and liver to stimulate bone marrow erythrocyte production. EPO and EPO receptors (EPO-R) are widely expressed in different organs including brain, spinal cord, retina (mainly retinal pigment epithelium, RPE), heart, gut, kidney, muscle, lung, and testis (Erbayraktar et al., 2003; Gassmann et al., 2003; Hernandez et al., 2006). EPO is the dominant HIF-1α target and acts as an initial factor to initiate the hypoxia condition as well as works independently without VEGF to promote neovascularization (Jaquet et al., 2002; Watanabe et al., 2005). VEGF is a HIF-1α responsive cytokine and is the primary contributor to mitosis and the development of abnormal vessel growth and angiogenesis. In a physiological setting, endogenous VEGF, VEGF-A in particular, is required for RPE stability as well as maintenance and function of adult retina neuronal cells, especially the survival of Müller cells and photoreceptors (Nishijima et al., 2007; Saint-Geniez et al., 2008). VEGF-associated complications are extensively described in retinal angiogenesis diseases. VEGF is highly expressed under hypoxia; its expression leads to retinal vessel leakage and retinal endothelial cell proliferation and migration, thereby resulting in neovascularization. Anti-VEGF therapy (bevacizumab or ranibizumab) is largely prescribed for treatment of angiogenesis and vessel permeability in wet AMD, DR and RVO (Moravski et al., 2003; Ray et al., 2004; Nagpal et al., 2007). HO-1, one target product of HIF-1α, is an antioxidative stress transcription factor and is regulated by antioxidant/electrophile response elements (ARE/EpRE) that scavenge heme to iron, carbon monoxide and biliverdin. HO-1 is also involved in the vasculature and angiogenesis of tumors, wounds and so on. ADM is a small, secreted multifunctional peptide expressed in various organs including the retina (Lopez and Martinez, 2002; Blom et al., 2012). ADM and its receptor are localized in neural tissues of the embryo (Montuenga et al., 1997) and are present throughout the retina of the adult mouse (Blom et al., 2012). Lack of a functional ADM gene resulted in embryonic lethality (Caron and Smithies, 2001; Shindo et al., 2001; Iesato et al., 2013). ADM heterozygous knockout mice exhibited reduced expression of VEGF and endothelial nitric oxide synthase (eNOS) during retinal angiogenesis, and ADM blockade reduced pathological retinal angiogenesis (Iesato et al., 2013). ADM is also involved in some inflammatory and proliferative ocular diseases whose pathogenesis is ischemia (Takahashi, 2001; Udono-Fujimori et al., 2003). Its expression is upregulated in response to hypoxic preconditioning in the retina (Udono et al., 2001; Thiersch et al., 2008; Zhu et al., 2008; Ponnaluri et al., 2011). It works together with VEGF to promote angiogenic effect in ischemia (Iimuro et al., 2004). Glucose is imperative for normal functioning of retinal neurons and glial cells as they have high-energy demands. Glucose entry into the retina is a critical part for retinal normoxia. Glucose transporter-1 (Glut-1), also known as solute carrier family 2, is an integral membrane glycoprotein and a sole glucose transporter between blood and retina (blood-retinal barrier, BRB). The aerobic glycolysis in the retina is reflected by the increased blood flow metabolite of Glut-1. Glut-1 is also one of the several adaptive metabolites including glucolytic enzymes and pyruvate dehydrogenase kinase, to allow retina to better respond to the ischemia condition. In cultured Müller glial cells, quantitative polymerase chain reaction (qPCR) revealed that Glut-1 mRNA expression was 5.85- and 116-fold greater than that of Glut-3 and -4, respectively (Hosoya et al., 2008), suggesting that Glut-1 is a dominant transporter in regulating glucose utilization.

The expression of HIF-1α target genes and the associated effects in the retina are presented in Table 1. Their neuroprotective role in retina, especially in the models of glaucoma, wet AMD and DR, is highlighted and discussed.

**Table 1:** **Effects of HIF-1α target genes on promoting retinal neuroprotection**.

**HIF-1α targets**	**Expression in the retina**	**Approximate molecular weight (in human)**	**Effects**	**References**
EPO	↑EPO in RPE than in neuroretina, ↑intravitreal EPO in diabetic patients than nondiabetic patients	34KD	Polycythemia, anti-apoptosis, neurotrophic, angiogenic effect	Garcia-Ramirez et al., 2008
VEGF-A	RPE, retinal capillaries, etc.	Monomer: 21KD; dimer: 42KD	Stimulate vasculogenesis and endothelial cell growth, promote permeabilization of blood vessels and cell migration, and inhibit apoptosis	Shima et al., 1995; Saint-Geniez et al., 2008
HO-1	Müller cells, RPE	32KD	Catalyze the degradation of heme, and therefore produce biliverdin, iron and carbon monoxide. Respond to oxidative stress, hypoxia, heavy metals, cytokines, and so on. Promote vasculature and angiogenesis	Choi and Alam, 1996; Kikuchi et al., 2005; Morse and Choi, 2005
ADM	Photoreceptor outer segments, ONL, Müller and amacrine cell somata in the INL, and some somata in the GCL	12167KD~6028.73KD, varies	Vasodilator, upregulate angiogenesis, increase tolerance to oxidative stress and hypoxic injury	Blom et al., 2012
Glut-1	RPE, choroidal, iridial and pars planus, BRB, Müller cell, lens, iris, photoreceptors	55KD	Facilitate the transport of glucose across BRB	Nihira et al., 1995
bFGF	Immature photoreceptors during postnatal development, photoreceptor outer segment/interphotoreceptor matrix complex, INL in adult retina	17.4KD	Wound healing, endothelial cell mitogen, mediate angiogenesis	Gao and Hollyfield, 1995
iNOS	Ganglion cells, INL and glial cells in diabetic eyes, microglia of the developing quail retina	150-160KD, divided into nNOS and eNOS	Synthesis of nitric oxide (NO), NO is a messenger involved in vasodilatation, neurotransmission, antimicrobial and anti-tumor activities	Abu El-Asrar et al., 2004; Sierra et al., 2014

*RPE, retinal pigment epithelium; ONL, outer nuclear layer; INL, inner nuclear layer; GCL, ganglion cell layer; BRB, blood-retinal barrier; nNOS, neuronal nitric oxide synthase; eNOS, endothelial nitric oxide synthase*.

*↑, increased*.

## HIF-1α and its target genes in models of retinal ganglion cell degeneration

Retinal ganglion cells (RGCs), located near the inner surface of the retina, receive visual information from photoreceptors and convey visual signals to the brain via their axons. Damage to the optic nerve and RGCs lead to permanent loss of vision. RGC degeneration models mimic the diseases of glaucoma, optic neuritis, traumatic optic neuropathy injury, and etc. HIF-1α was detected in the retina and optic nerve head of human glaucomatous eyes compared with the control eyes, and its expression was closely concordant with the location of visual field defects recorded in these eyes (Tezel and Wax, 2004). These observations provide direct evidence that tissue hypoxia is present in the retina and optic nerve head of human glaucomatous eyes, and hypoxic signaling is a component of the pathogenic mechanisms of glaucomatous neurodegeneration.

The dominant HIF-1α target EPO is well-defined in protecting against ischemic brain injury in the CNS (Marti et al., 2000; Ehrenreich et al., 2002; Kretz et al., 2005). In the retina, endogenous EPO protects neuroretinal function in ischemic retinopathy. Böcker-Meffert et al. first reported that EPO could promote neural outgrowth of axotomized RGCs in a dose-dependent manner (Böcker-Meffert et al., 2002). Systemic administration of recombinant human EPO (rhEPO) before or immediately after retinal ischemia was found to not only reduce histopathological damage and promote functional recovery in electroretinography, but also decrease apoptotic neurons (Junk et al., 2002). Sullivan et al. showed that systemic administration of adeno-associated viruses (AAVs)–mediated mutant form of EPO (EPOR76E) preserved RGCs and visual function in DBA/2J glaucomatous mice. The rescued RGCs retained their axonal projections within the optic nerve and the hematocrit did not exceed normal limits (Sullivan et al., 2011). Retrobulbar administration of EPO also protected RGCs from acute elevated intraocular pressure (IOP; Zhong et al., 2008) and axotomy (Kilic et al., 2005). The survival rate of the RGCs 14 days after axotomy lesion was markedly increased from about 20% to more than 60% (Kilic et al., 2005), which surpasses the neuroprotection efficacy of classical neurotrophins, such as brain-derived neurotrophic factor (BDNF; Klocker et al., 2000), insulin-like growth factor-1 (IGF-1; Kermer et al., 2000) or glial cell line-derived neurotrophic factor (GDNF; Kilic et al., 2004). Consistent with these notions, systemic applied recombinant EPO protected EPO-deficient mice against profound retinal ischemia damage (Mowat et al., 2012) in RGCs (Junk et al., 2002). And subretinal transplantation of EPO gene-modified rat mesenchymal stem cells (rMSCs) worked better than rMSCs alone for sodium iodate (SI)-induced retinal degeneration in rats (Guan et al., 2013). Taken together, the neuroprotective role of EPO in RGCs is clear and is mainly accomplished by its anti-apoptosis (Chung et al., 2009), neurotrophic, and angiogenic effects.

VEGF, another HIF-1α target, was depicted to have protective roles for non-vascular cells in various models (Yang and Cepko, 1996; Coultas et al., 2005; Maharaj and D'Amore, 2007; Emerich et al., 2010). For instance, some isotypes of VEGF displayed neuroprotective properties *in vitro* (Jin et al., 2000). *In vivo* studies showed that endogenous VEGF-A165b isoform exerted neuroprotective effects in response to glutamatergic excitotoxicity on RGCs during rat retinal ischemia-reperfusion injury, through activation of VEGFR-2 and MEK1/2 pathways and inhibition of caspase-3 (Beazley-Long et al., 2013). VEGF-A promoted glaucomatous RGC survival via VEGFR-2, thereby highlighting the potential risks associated with using VEGF-A antagonists in ocular diseases (Foxton et al., 2013). Anti-VEGF reduced the potential benefit of VEGF in neuroprotection and exacerbated RGC apoptosis (Nishijima et al., 2007). Similar to EPO, VEGF protects axotomized RGCs from degeneration through ERK1/2 and Akt pathways (Böcker-Meffert et al., 2002; Kilic et al., 2006). Ischemic preconditioning prior to ischemia-reperfusion injury showed increased VEGF-A levels. And the VEGF-A levels substantially decreased in a dose-dependent manner following RGC apoptosis. Despite the overwhelming evidence indicating a role for VEGF in neuroretinal protection, a few groups did not observe any damage to retinal photoreceptors or RGCs after blocking VEGF or its receptors (Miki et al., 2010; Sobaci et al., 2013; Demirel et al., 2015). These discrepancies suggest a possibility that the neuroprotective function exerted by VEGF may be conditional and context-dependent. Therefore, further studies to clarify the neurotrophic role of VEGF in RGCs is warranted.

Oxidative stress is considered to be an early event of various retinal diseases (Liu et al., 2007). And indeed, HO-1 exerts a protective effect on ocular diseases. Previous findings suggested that functional human HO-1 could prevent RGC death in the adult rat retina after pressure-induced ischemia (Hegazy et al., 2000). The nuclear factor erythroid 2-related factor (Nrf2)/HO-1-antioxidant pathway was activated in ischemia-reperfusion-induced rat retinal damage, and this activation led to better preservation of ganglion cell layer (GCL) and inner nuclear layer (INL; Varga et al., 2013; He et al., 2014). Nrf2 and HO-1 were also increased in attenuated GCL damage following limb remote ischemic conditioning of the retina (Zhang X. et al., 2014). Overexpression of HO-1 in RGCs facilitated the survival of some RGCs for up to 7 days after optic nerve crush (Himori et al., 2014). AAV-mediated HO-1 gene transfer (AAV-HO-1) into the vitreous promoted RGC survival following ischemia/reperfusion-induced damage, while inhibiting HO-1 counteracted the effect in rats (Peng et al., 2008). Stimulation of Nrf2/HO-1 axis could be of interest for the treatment of retinal degeneration such as glaucoma, AMD, and DR which will be discussed below (Foresti et al., 2013, 2015). Furthermore, increased ADM was found in the vitreous humor of glaucoma (Evereklioglu et al., 2002), DR (Ito et al., 2003; Shaw et al., 2006; Weng et al., 2006), uveitis, and vitreoretinal patients (Udono et al., 2002), and in the plasma of retinitis pigmentosa individuals (Vingolo et al., 2005). Glucose deprivation may result in an increased ability to protect RGCs from glutamate-induced excitotoxicity (Toft-Kehler et al., 2014), but the function of Glut-1 was not tested in the RGC degeneration models.

The efficacy of HIF-1α and its target genes in protecting against RGC degeneration may open new perspectives for the treatment of glaucoma, optic neuritis, and traumatic optic neuropathy injuries. Developing new treatment targeting on HIF-1α target genes for RGC degeneration deserves concerted efforts in the near future.

## HIF-1α and its target genes in models of photoreceptor degeneration and wet AMD

The pathogenesis of AMD is not well-known. The wet AMD, also known as neovascular or exudative AMD, causes central vision loss due to RPE atrophy, neovascularization, and death of photoreceptors. Oxidative stress was implicated in the senescence of RPE cells and the pathogenesis of AMD. Development of choroid neovascularization (CNV), which is mediated mainly by HIF-1α and VEGF, is the most significant threat for AMD; therefore, inhibiting HIF-1α and VEGF is often used for treating AMD. However, it was found that focused activation of HIF transcription factors in normoxic photoreceptors resulted in a transient protection of rods against light damage (Lange et al., 2011). Pyruvate application, which stabilized HIF-1α and HIF-2α, protected the mouse retina against white light damage (Ren et al., 2011).

Unlike HIF-1α, the effect of EPO on protecting photoreceptors and RPE cells is uncontroversial. EPO is generally regarded as a potent neuroprotective factor for photoreceptors (Becerra and Amaral, 2002). In the adult mammalian retina, systemically applied EPO was protective against light-induced photoreceptor apoptosis (Becerra and Amaral, 2002; Grimm et al., 2002). It stabilized the retinal vasculature and inhibited the development of focal vascular lesions in photoreceptor degeneration (Shen et al., 2014). Subretinal delivery of EPO protected against light-induced and genetic photoreceptor degeneration (Zhang et al., 2008; Rex et al., 2009; Colella et al., 2011; Busch et al., 2014) through the ERK1/2 and Akt pathway (Shen et al., 2010). An earlier report revealed that by either transgenic or systemic applications, EPO protected against apoptotic cell death during acute, light-induced photoreceptor cell death but not in genetically based retinal degeneration such as in retinal degeneration 1 (rd1) or dominant retinitis pigmentosa mouse models (Grimm et al., 2004). Findings from a study demonstrated that subretinal injection of EPO AAVs resulted in approximately an 8 μm thicker outer nuclear layer (ONL) in retinas of retinal degeneration slow (rds) mice compared to the control group (Hines-Beard et al., 2013). Nevertheless, one may argue that the neuroprotective effect of EPO is short-lived and the ONL may degenerate without a repeated hypoxia preconditioning (Grimm et al., 2005). For RPE cells, EPO, and EPO-R are highly enriched in light-induced apoptotic RPE cells (Chung et al., 2009). EPO protected RPE from oxidative damage by decreasing inflammatory cytokines of tumor necrosis factor-α (TNF-α) and interleukin-1β (IL-1β), and inhibiting caspase-3-like activity (Gawad et al., 2009; Wang et al., 2009).

Anti-VEGF agents are widely used in the treatment of choroidal neovascularization for AMD. However, long-term VEGF inhibition could have deleterious effects on the remaining healthy retina given the well-documented role of VEGF in normal development of the retinal vasculature (Hernandez and Simo, 2012). Non-isoform-specific inhibition of VEGF for treating angiogenesis can constrain the neuroprotective role of some isoforms of VEGF and damage retinal and sensory neurons. Inhibiting VEGF following photodynamic therapy (PDT) which causes elevated VEGF resulted in photoreceptor apoptosis, suggesting that VEGF after PDT is neuroprotective (Suzuki et al., 2011). Depletion of VEGF-A specifically in neural progenitor cells resulted in thinner retina and aberrant cortex development (Haigh et al., 2003).

Expression of HO-1 in RPE is essential for the survival of photoreceptors (Satarug et al., 2008) and increases following repetitive hypoxia preconditioning in the retina (Zhu et al., 2007). HO-1 is also a sensitive marker for light-induced insult in the retina (Kutty et al., 1995). An increased expression of HO-1 is thought to be a cytoprotectant against light-induced oxidative damage in the retina (Kutty et al., 1995). HO-1 overexpression protected photoreceptors from cellular damage caused by intense light exposure (Sun et al., 2007) and protected RPE cells against the toxic effect induced by high glucose and oxidative/nitrosative stress (Castilho et al., 2012). RPE cells derived from neovascular AMD patients displayed much higher HO-1 and HO-2 antigen levels compared to those from younger individuals, suggesting that HO has protective mechanisms against oxidation (Frank et al., 1999).

ADM, another HIF-1α target, is the first neurotransmitter/neuromodulator found in the RPE (Udono et al., 2000, 2002). It can promote hypoxia vasodilation in retinal arteries (Maenhaut et al., 2007). Additionally, ADM suppressed the migration, proliferation and tube formation of human RPE cells under hypoxia (Chen et al., 2014). Administration of ADM in mice inhibited macrophage migration from RPE to prevent choroidal neovascularization (Yuda et al., 2012). These results were in agreement with a neuroprotective role for ADM in ischemic brain injury (Willis et al., 1996; Dogan et al., 1997; Xia et al., 2004, 2006; Miyashita et al., 2006; Igarashi et al., 2014; Zhang H. et al., 2014).

The above evidence suggests that HIF-1α and its target genes can act as a retinal shield against photoreceptor degeneration and wet AMD injuries in the retina. Further studies are required to clarify the role of these transcription factors in retinal pathology to determine whether they can be exploited as potential therapeutic targets in treating photoreceptor degeneration and wet AMD.

## HIF-1α and its target genes in models of diabetic retinopathy

Elevated HIF-1α and VEGF are generally accepted to be harmful for diabetic retinopathy, despite some studies found that anti-VEGF in DR rats had detrimental effects on neuronal cells (RGC, amacrine and bipolar cells) which are present in the inner layers of the retina (Park et al., 2014). The situation is different for the HIF-1α targets such as EPO, HO-1, ADM, and Glut-1. Strikingly high levels of intravitreal EPO have been found in both proliferative diabetic retinopathy (PDR) and diabetic macular edema (DME) patients (Hernandez et al., 2006). EPO-R is upregulated in DR neurosensory retina in response to diabetic stress (Junk et al., 2002; Zhang et al., 2008). The upregulation of EPO/EPO-R leads to a maintenance-survival mechanism thereby adapting to insulin increase in early DR. Intravitreal injection of EPO induced downregulation of EPO-R, VEGF, and VEGF receptor in streptozocin-induced DR rats (Mitsuhashi et al., 2013). In DR, EPO-derived peptide and EPO helix-B domain significantly protected against neuroglial and vascular degeneration without altering hematocrit or exacerbating neovascularization or thrombosis in diabetic rats (McVicar et al., 2011). Administering suberythropoietic EPO is also vaso- and neuro-protective in experimental early DR both *in vivo* and *in vitro* (Zhang et al., 2008; Wang et al., 2011; Mitsuhashi et al., 2013).

HO-1 was found to be increased in the retina of db/db DR mice and cultured retinal explants (He et al., 2013). It was reported that HIF-1α-mediated, long-lasting HO-1 elevation contributed to long-term retinal ischemia tolerance (Zhu et al., 2007). Induction of HO-1 by compounds or oxidative stress reduced damage in the retina (Mandal et al., 2009; Kim et al., 2012; Chao et al., 2013; Rappoport et al., 2013; Koskela et al., 2014; Lee et al., 2014) and decreased retinal microvascular complications in DR (Geraldes et al., 2008) through the IRS1/PI3K/Akt2 pathway (Cukiernik et al., 2003). Overexpression of HO-1 in the retina restored visual function in diabetic rat models (Geraldes et al., 2008; Shyong et al., 2008) and systemic hypoxia mice (Zhu et al., 2007). Furthermore, diminished HO-1 expression in RPE cells was found in diabetic patients (da Silva et al., 1997). HO-1 also exerts anti-inflammatory, anti-apoptotic and anti-proliferative effects through Nrf2/ERK-related signaling (Peng et al., 2011; Fan et al., 2012). It could drive the resolution of inflammation by reducing macrophage infiltration in the ischemia-reperfusion injured retina (Sun et al., 2010). Other *in vitro* experiments supported a similar anti-inflammatory role for HO-1 (Willis et al., 1996). Inhibition of upregulated HO-1 in Müller cells resulted in increased infiltration of inflammatory cells and destruction of the retina in ischemia-reperfusion injury rats (Ulyanova et al., 2001; Arai-Gaun et al., 2004). Inhibition of HO-1 also enhanced reactive oxygen species (ROS) production and the toxic effect (Castilho et al., 2012). These findings have revealed that HO-1 is cytoprotective by helping the cells to adapt to the oxidative stress environment.

For ADM, its plasma level was lower in juvenile type 1 diabetes patients without retinopathy than that in healthy subjects (Semeran et al., 2013). Glut-1, as indicator of glucose transportation in the retina, played a big role in the pathophysiology of DR. In the human and monkey, Glut-1 is diffusely expressed throughout the whole retina, including the BRB cells, RPE, vascular endothelium, and rod and cone inner and outer segments (Nihira et al., 1995). Elevated levels of Glut-1 increased the ability of retina to utilize glucose for normal metabolic activity (Schubert, 2005). Knockdown or blockade of Glut-1 by small interfering RNAs (siRNAs) significantly reduced retinal glucose in diabetic mice (Lu et al., 2013). In contrast, some studies suggested that inhibiting Glut-1 could prevent expression of early biomarkers of DR (Lu et al., 2013) and that Glut-1 was downregulated in oxidative RPEs (Fernandes et al., 2011). Expression of Glut-1 in the microvasculature does not account for the glucose accumulation in diabetic retina (Puchowicz et al., 2004). Therefore, the neuroprotective function of Glut-1 in the retina needs to be further investigated.

## Discussion

Some other HIF-1α targets such as basic fibroblast growth factor (bFGF; Cuevas et al., 1998; Yu et al., 2004; Valter et al., 2005), inducible nitric oxide synthase (iNOS; Sakamoto et al., 2006; Zhu et al., 2006; Sun et al., 2010), etc. which are less reported in retinal neuroprotection are not discussed here. The selected reports of HIF-1α target genes on retinal neuroprotection are summarized in Table 2.

**Table 2:** **Selected reports of HIF-1α target genes involved in retinal neuroprotection**.

**HIF-1α targets**	**Interventions**	**Observed objects**	**Findings**	**Conclusions**	**References**
EPO	Systemic administration of recombinant human EPO (rhEPO)	Transient global retinal ischemia induced by raising IOP	↑EPO-R in the retina, EPO with soluble EPO-R exacerbated ischemic injury, ↓histopathological damage, ↑functional recovery in ERG	Exogenous EPO has an antiapoptotic mechanism of action; EPO is as a neuroprotective agent in acute neuronal ischemic injury	Junk et al., 2002
	Treat with EPO and VEGF	Retinal explants from postnatal rats	EPO and VEGF improved neurite outgrowth of RGCs	EPO and VEGF have a significant and specific biological effect on neurite regrowth of axotomized RGCs; EPO and VEGF have a neuroprotective and neuroregenerative role on RGCs in ischemic retina	Böcker-Meffert et al., 2002
	*In vitro* culture under glycosylated insult	Chemical-induced insults in primary retinal neurons	EPO was shown to be antiapoptotic, ↑Bcl-XL and p-BAD, ↓Bax	EPO/EPO-R acts through ERK-1/2 and Akt pathways	Shen et al., 2010
	Damage from ROS	Oxidant-treated cultured human RPE cells	EPO ↑RPE cells viability, ↓inflammatory cytokines TNF-α and IL-1β, ↓cell DNA fragmentation and membrane phosphatidylserine exposure, ↓ROS, ↓caspase-3	EPO protects against oxidative injury-induced cell death and mitochondrial dysfunction in RPE cells through modulation of p-Akt1 and mitochondrial membrane potential	Wang et al., 2009
	Optic nerve transection	*In vivo* retrograde degeneration of RGCs in mouse line tg21 that constitutively expresses human EPO	↓p-STAT-5, Bcl-XL in RGCs, ↑p-ERK-1/2 and p-Akt, ↓caspase-3	Predict clinical implementation of recombinant human EPO not only in patients with acute ischemic stroke but also with more delayed degenerative neurological diseases	Kilic et al., 2005
	EPO single intravitreal injection	Diabetic Sprague-Dawley rats	↑EPO-R in the neurosensory retina, improvement of photoreceptor survival, but endogenous EPO in neurosensory retina was unchanged, activation of the ERK but not the STAT-5 pathway	EPO/EPO-R is a maintenance-survival mechanism of retinal neurons; responds to the insults of early diabetes other than ischemia; Intravitreally injection of EPO in early diabetes may prevent retinal cell death and protect the BRB function	Zhang et al., 2008
	EPO intravitreal injection	Photoreceptor degeneration in retinal detachment rat	↓caspase-3, ↑Bcl-XL, anti-apoptosis of photoreceptors; ↑p-JAK2, p-Akt and p-ERK-1/2 by 400ng EPO treatment	Intravitreal injection of 400ng EPO is safe and photoreceptor-protetive; EPO may activate PI3K/Akt and MAPK/ERK-1/2 pathways	Xie et al., 2012a,b
	Acute hypoxia-induced EPO; systematic administration of EPO	Photoreceptor degeneration by light and surgery in mice or rats	↓caspase-1, ↑EPO-R in photoreceptors; stabilized the retinal vasculature, ↓photoreceptor apoptosis, ↑CD34^+^ cells into the retina	EPO protects photoreceptors through ↓p75NTR-pro-NT3 signaling, ↑production and mobilization of bone marrow derived cells	Grimm et al., 2002; Shen et al., 2014
VEGF	Intravitreal anti-VEGF-A antibody injection	Streptozotocin-induced diabetic rat retina	↑RGC death, novel apoptosis in amacrine and bipolar cells, ↓p-Akt	The p-Akt pathway, which plays a neuroprotective role via VEGF, was significantly affected by VEGF inhibition; ↓VEGF may have detrimental effects on neuronal cells	Park et al., 2014
	Axotomy	A transgenic mouse line that constitutively expresses human VEGF	RGCs of VEGF- transgenic mice were protected against delayed degeneration after axotomy; ↑p-ERK-1/2, ↑p-Akt, ↓p38, ↓caspase-3	VEGF exerts neuroprotection by dual activation of ERK-1/2 and Akt pathways	Kilic et al., 2006
	VEGF-A; VEGF-A165b treatment	Rat glaucoma model or ischemia-reperfusion injury in rats or *in vitro* retina culture or RGC insults in rat retinal ischemia-reperfusion injury	Dose-dependent↓ in retinal neuron apoptosis, ↑VEGFR-2; VEGF-A acts directly on RGC to promote survival, VEGFR-2 signaling via the pathway of phosphoinositide-3-kinase/Akt, VEGF-A protects RGC via VEGFR-2; neuroprotective through activation of VEGFR-2 and MEK-1/2, not via p38 activation, ↓caspase-3	Antagonism of VEGF-A function presents a risk to neuronal survival; VEGF-A165b may be therapeutically useful for pathologies that involve neuronal damage, non-isoform-specific inhibition of VEGF-A may be damaging to retinal and sensory neurons	Nishijima et al., 2007; Beazley-Long et al., 2013; Foxton et al., 2013
	siRNA-based ↓ VEGF	Müller cells and photoreceptors	↓INL and ONL thickness, ↓retinal function in ERG	Endogenous VEGF is required for visual function	Saint-Geniez et al., 2008
HO-1	↑HO-1 in photoreceptors by AAV gene subretinal injection	Light-injured Sprague-Dawley rats	Partially preserved retina structure and attenuated apoptosis in photoreceptors, ↓c-fos, ↓p53, ↑p38, ↑bcl-2, ↑c-FLIP	The anti-apoptotic mechanisms of HO-1 may be related to ↑p38, bcl-2 and c-FLIP and ↓c-fos and p53	Sun et al., 2007
	Insulin-induced HO-1 treatment	Bovine retinal endothelial cells (BREC) and pericyte cells (BRPC) from fresh calf eyes	Insulin-induced HO-1 through PI3-kinase/Akt pathway without affecting ERK and p38 MAPK; insulin regulated HO-1 expression via IRS1 and Akt2 pathways; ↓NF-κB, ↓caspase-8 and apoptosis via the IRS1/PI3K/Akt2/HO-1 pathway	Insulin activates HO-1 expression via IRS1/PI3K/Akt2 signaling	Geraldes et al., 2008
ADM	Culture under normoxic or hypoxic conditions	RPE cells	Hypoxia ↑ADM in all three human RPE cell lines, ADM treatment ↓the hypoxia-induced cell number decrease	ADM induced by hypoxia protects cell damage in RPE cells	Udono et al., 2001
	Oxygen-induced retinopathy	Heterozygous KO mouse of ADM [ADM(+/−)] and its receptors, inducible endothelial cell-specific RAMP2 KO mouse line [DI-E-RAMP2(−/−)]	↓VEGF and eNOS in ADM(+/−) retinas, DI-E-RAMP2(−/−) showed abnormal retinal vascular patterns in the early stages of development, ADM enhanced the proliferation and migration of RPE cells, intravitreal injection of anti-ADM antibody ↓pathological retinal angiogenesis	The ADM-RAMP2 system is crucially involved in retinal angiogenesis; ADM and its receptor system are potential therapeutic targets for controlling pathological retinal angiogenesis	Iesato et al., 2013
Glut-1	Knockdown Glut-1 by siRNA and systemic administration of Glut-1 inhibitor	Diabetic mice	↓Retinal glucose by ↓Glut-1; Glut-1 inhibitors reduced glucose and glycohemoglobin levels in RBC, prevented early biomarkers of DR including superoxide radicals, chaperone protein β2 crystallin and VEGF	Anti-Glut-1 treatment is a promising therapeutic target for preventing DR	Lu et al., 2013
	Diabetics induced by streptozotocin	Streptozotocin-induced diabetic rats vs. nondiabetic rats (normoglycemic and acute-hyperglycemic)	Retinal glucose influx in the diabetic rats was lower than in the nondiabetic acute-hyperglycemic group, but not in the normoglycemic group, ↑glucose in the diabetic retina than the nondiabetic retina	The accumulation of glucose in the diabetic retina cannot be explained by increased endothelial-glucose uptake (Glut-1)	Puchowicz et al., 2004
bFGF	Genetic degeneration of photoreceptors	Photoreceptor degeneration rd mice	↑bFGF in the outer retina during photoreceptor degeneration, weakly present in some cells in the INL	Neuronal degeneration is accompanied by ↑bFGF in degenerating neurons prior to cell death	Gao and Hollyfield, 1995
iNOS	Ischemia preconditioning	WT mice *vs*. WT mice treated with NOS inhibitor before preconditioning *vs*. iNOS/eNOS/nNOS KO mice	Ischemic tolerance was not achieved in the retinas of NOS KO mice; NOS inhibitor to WT mice blocked the development of ischemic tolerance	NO derived from both eNOS and nNOS is a required molecular signal in the adaptive response to ischemic preconditioning in the retina	Zhu et al., 2006

*ERG, electroretinography; Bcl-XL, B-cell lymphoma-extra large; p, phosphorylation; BAD, Bcl2-associated agonist of cell death; Bax, BCL2-associated x protein; ERK, extracellular signal-regulated kinase; Akt, Akt serine/threonine kinase; STAT, signal transducer and activator of transcription; JAK2, Janus kinase 2; PI3K, phosphoinositide 3-kinase; MAPK or MEK, mitogen-activated protein kinase; p75NTR-pro-NT3, p75 neurotrophin receptor-pro-mature neurotrophin-3; c-fos, fos proto-oncogene; bcl-2, B-cell lymphoma-2; c-FLIP, cellular FLICE (FADD-like IL-1β-converting enzyme)-inhibitory protein; IRS1, insulin receptor substrate 1; NF-κB, nuclear factor-κB; DI-E-RAMP2(−/−), drug-inducible vascular endothelial cell-specific RAMP2 knockout (−/−) mice; RAMP2, receptor activity modifying protein 2; RBC, red blood cells; WT, wildtype; KO, knockout; iNOS, inducible nitric oxide synthase; eNOS, endothelial nitric oxide synthase; nNOS, neuronal nitric oxide synthase; ↑, promote or increase or induce; ↓, inhibit or decrease or suppress. rd mouse is a spontaneous mouse mutant in which photoreceptors degenerate shortly after birth*.

HIF-1α together with its target genes are essential for retinal development, vasculature stability, proper retinal function, and vision maintenance. Hypoxia pre-conditioning mimics the physiopathological responses of the retina to provide protection for neuronal cells. PHD inhibitors stabilize HIF transcription factors, especially HIF-1α, to mediate genetic adaptation to hypoxia. Under hypoxia, the neuroprotective effect of HIF-1α targets might be multimodal, including promotion of oxygen and glucose supply, neovascularization, antioxidization, anti-inflammation, anti-apoptosis, and neurotrophy.

Nevertheless, the HIF-1α target gene therapy may be a double-edged sword (Grimm and Willmann, 2012). High-dose toxicity and longtime exposure may over-activate HIF-1α and bring about severe consequences in terms of side effects. Several lines of evidence have suggested that HIF-1α overexpression can contribute to retinal pathologies, including retinopathy of prematurity, DR, AMD, glaucoma, and high altitude retinopathy (Arjamaa and Nikinmaa, 2006; Willmann et al., 2013). It may also promote neovascularization, oncogenesis, and metastasis. New method seeking to manipulate the HIF-1α target genes to treat various ocular diseases as well as to reduce the side effects are warranted for new therapeutic avenues. Take the dominant HIF-1α target EPO as an example. Accumulating evidence makes EPO particularly attractive in preventing retinal degeneration in the early stages of retinal diseases. However, before applying EPO for retinal degenerative diseases, two critical points need to be taken into account. The first is the EPO form. Longtime usage and overdose of EPO lead to chronic polycythemia, which is a lethal side effect (Gassmann et al., 2003). Engineering an effective package for neuroprotective EPO and attenuating erythropoietic and angiogenic activity is the key for such a therapy. Secondly, timing and dose are crucial for EPO treatment of retinal diseases. It has been shown that intraocular delivery of EPO has a lower efficiency than when administered systemically. This suggests that hormone systemic effects contribute to progressive neuroprotection (Colella et al., 2011). Furthermore, high levels of EPO is not protective due to its bell-shaped dose curve (Hines-Beard et al., 2013), and the neuroprotective dose is higher than that required for erythropoiesis (Coleman and Brines, 2004). Early retinal EPO replenishment improves retinal vascular stability, but elevated EPO levels during the proliferation stage contribute to neovascularization and ocular diseases (Coleman and Brines, 2004; Chen et al., 2008). Therefore, early administration of EPO at a proper dose for retinal degeneration is important, for it can activate endogenous EPO-R in the affected tissue through stimulation of potent ischemic preconditioning. The treating course and strategy should be tailored according to patients' responses. Continuous delivery of EPO may lead to elevated local EPO and cause insults. Overall, timing, dose and route of administration are the important factors to be considered for balancing favorable *vs*. detrimental effects during EPO retinal therapy. Similar problems may be seen in exploiting other HIF-1α targets for treating retinal degeneration.

HIF-1α being a “master switch” for regulating all oxygen-dependent retinal diseases is critical in construction of new therapeutic avenues for treating retractable retinal insults. Additional preclinical experiments need to be conducted in order to elucidate the physiological and neuroprotective role of HIF-1α targets in the retina. A more refined understanding of the complex functions of HIF-1α and target genes will help to manipulate these genes and define new and more-specific targets for retinal degenerative diseases.

## Author contributions

LC and MX conceived the review. LC wrote the manuscript. HY, NY, and KL joined discussions and revised the paper. MX made critical revision and language editing. The manuscript has been reviewed and approved by all named authors.

### Conflict of interest statement

The authors declare that the research was conducted in the absence of any commercial or financial relationships that could be construed as a potential conflict of interest.

## Abbreviations

HIF: Hypoxia-inducible factor
EPO: erythropoietin
VEGF: vascular endothelial growth factor
HO-1: heme oxygenase-1
ADM: adrenomedullin
Glut-1: glucose transporter-1
CNS: central nervous system
AMD: age-related macular degeneration
DR: diabetic retinopathy
RVO: retinal vein occlusion
bHLH: helix-loop-helix
PHD: prolyl-hydroxylases
VHL E3: von Hippel-Lindau E3 ubiquitin ligase
ARNT: aryl hydrocarbon receptor nuclear translocator
HREs: HIF-responsive elements
EPO-R: EPO receptors
RPE: retinal pigment epithelium
ARE/EpRE: antioxidant/electrophile response elements
eNOS: endothelial nitric oxide synthase
BRB: blood-retinal barrier
qPCR: quantitative polymerase chain reaction
RGCs: retinal ganglion cells
rhEPO: recombinant human EPO
AAVs: adeno-associated viruses
IOP: intraocular pressure
BDNF: brain-derived neurotrophic factor
IGF: insulin-like growth factor
GDNF: glial cell line-derived neurotrophic factor
rMSCs: rat mesenchymal stem cells
SI: sodium iodate
Nrf2: erythroid 2-related factor
GCL: ganglion cell layer
INL: inner nuclear layer
CNV: choroid neovascularization
rd1: retinal degeneration 1
ONL: outer nuclear layer
rds: retinal degeneration slow
TNF-α: tumor necrosis factor-α
IL-1β: interleukin-1β
PDT: photodynamic therapy
PDR: proliferative diabetic retinopathy
DME: diabetic macular edema
ROS: reactive oxygen species
siRNAs: small interfering RNAs
bFGF: basic fibroblast growth factor
iNOS: inducible nitric oxide synthase.
